# A case of idiopathic gastroesophageal submucosal hematoma in a patient with no predisposition to bleeding

**DOI:** 10.1002/deo2.284

**Published:** 2023-08-21

**Authors:** Takaaki Iwado, Hirokazu Honda, Tatsuhiro Gotoda

**Affiliations:** ^1^ Department of Gastroenterology and Hepatology Kurashiki Central Hospital Okayama Japan

**Keywords:** esophageal hematoma, esophageal intramural hematoma, esophageal submucosal hematoma, gastroesophageal hematoma, gastrointestinal hematoma

## Abstract

Gastroesophageal submucosal hematoma is a disease in which blood vessels in the gastroesophageal submucosa rupture and form a hematoma. In this report, we describe a case of gastroesophageal submucosal hematoma that developed due to vomiting in a patient with no history of bleeding and resolved with conservative treatment. A 69‐year‐old man presented with precordial pain and hematemesis after vomiting. A diagnosis of idiopathic gastroesophageal submucosal hematoma was made by computed tomography scan, magnetic resonance imaging, and esophagogastroduodenoscopy. Healing was achieved by conservative treatment with fasting, rehydration, and acid suppression. When a patient presents with sudden chest pain, hematemesis, and dysphagia, the possibility of this disease should be considered.

## INTRODUCTION

Gastroesophageal submucosal hematoma is a rare condition characterized by the rupture of blood vessels within the gastroesophageal submucosa resulting in the formation of a hematoma. It commonly occurs as an incidental complication of endoscopic procedures and primarily affects individuals with coagulopathies or those taking antithrombotic medications. We report a case of gastroesophageal submucosal hematoma that developed due to nausea in the absence of coagulopathy and antithrombotic medication.

## CASE REPORT

A 69‐year‐old man experienced nausea after dinner. He vomited approximately 500 ml of dark red emesis. Gradually, he developed precordial pain, which prompted him to seek medical attention at our hospital. He had a medical history of type 2 diabetes mellitus, hypertension, and hyperlipidemia, for which he had been receiving insulin therapy for type 2 diabetes mellitus and other medications, but no antithrombotic medications.

**FIGURE 1:**
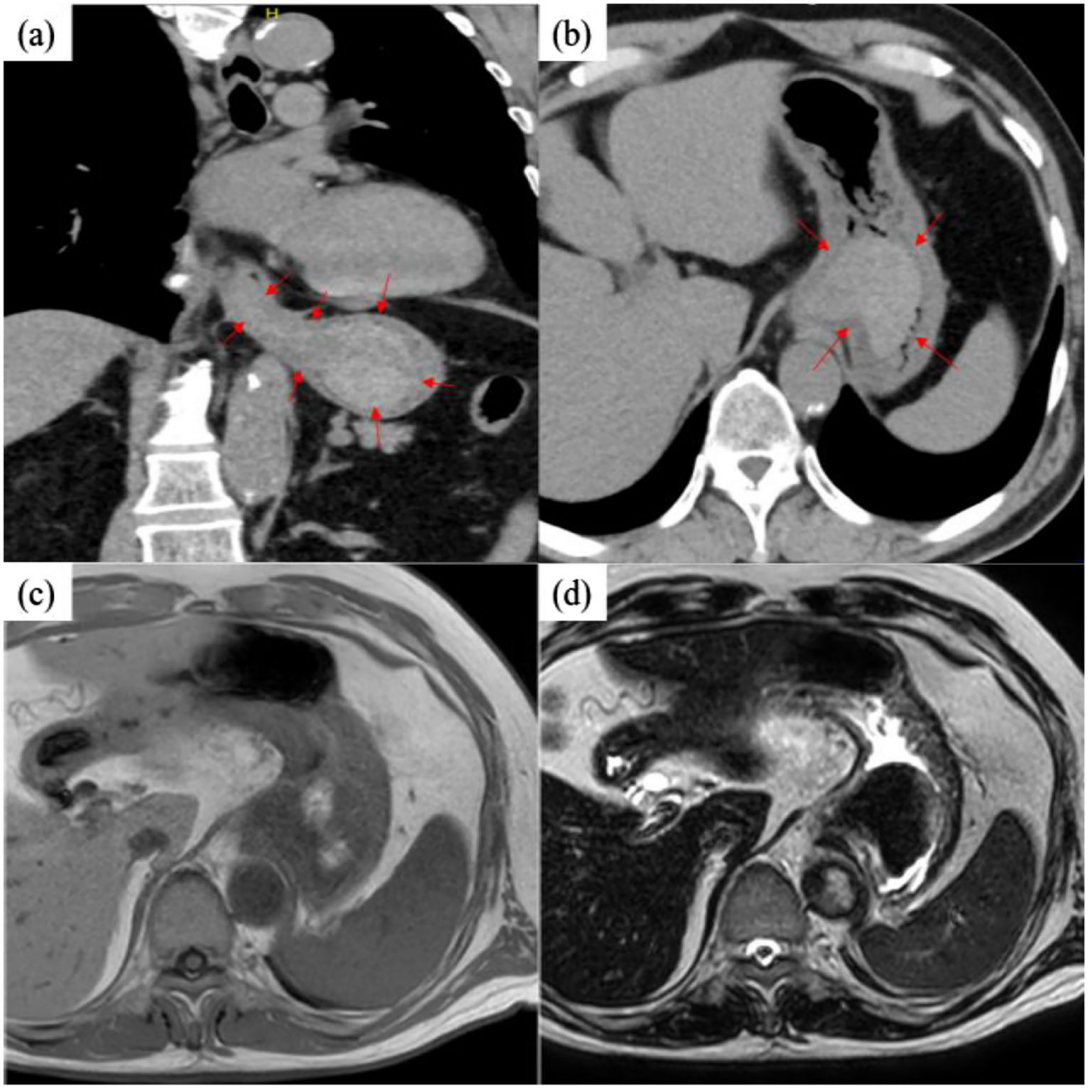
(a) Admission plain computed tomography scan showed a continuous hyperabsorptive zone from the thoracic esophagus to the stomach. (b) In the stomach, a clearly demarcated hyperabsorptive zone was observed from the fundus to the upper gastric body, which was considered to be a hematoma. (c) Magnetic resonance imaging on day 2 showed a pale high signal area in the stomach on T1‐weighted images. (d) T2‐weighted images also showed a low signal area, leading to the diagnosis of acute to subacute submucosal hematoma.

On arrival, his vital signs were within normal ranges. Blood tests showed a slight decrease in the serum hemoglobin level to 12.7 g/dL and a slight increase in the blood urea nitrogen level to 32 mg/dL. Platelet count, prothrombin time, and activated partial thromboplastin time were all within normal ranges. Due to his allergic reaction to iodine‐based contrast, a plain computed tomography (CT) scan was performed to identify the source of the bleeding, which revealed a suspected blood‐filled gastric reservoir and a suspected submucosal hematoma extending from the thoracic esophagus to the stomach (Figure 1a,b). Consequently, he was admitted to the hospital for further evaluation and treatment.

**FIGURE 2:**
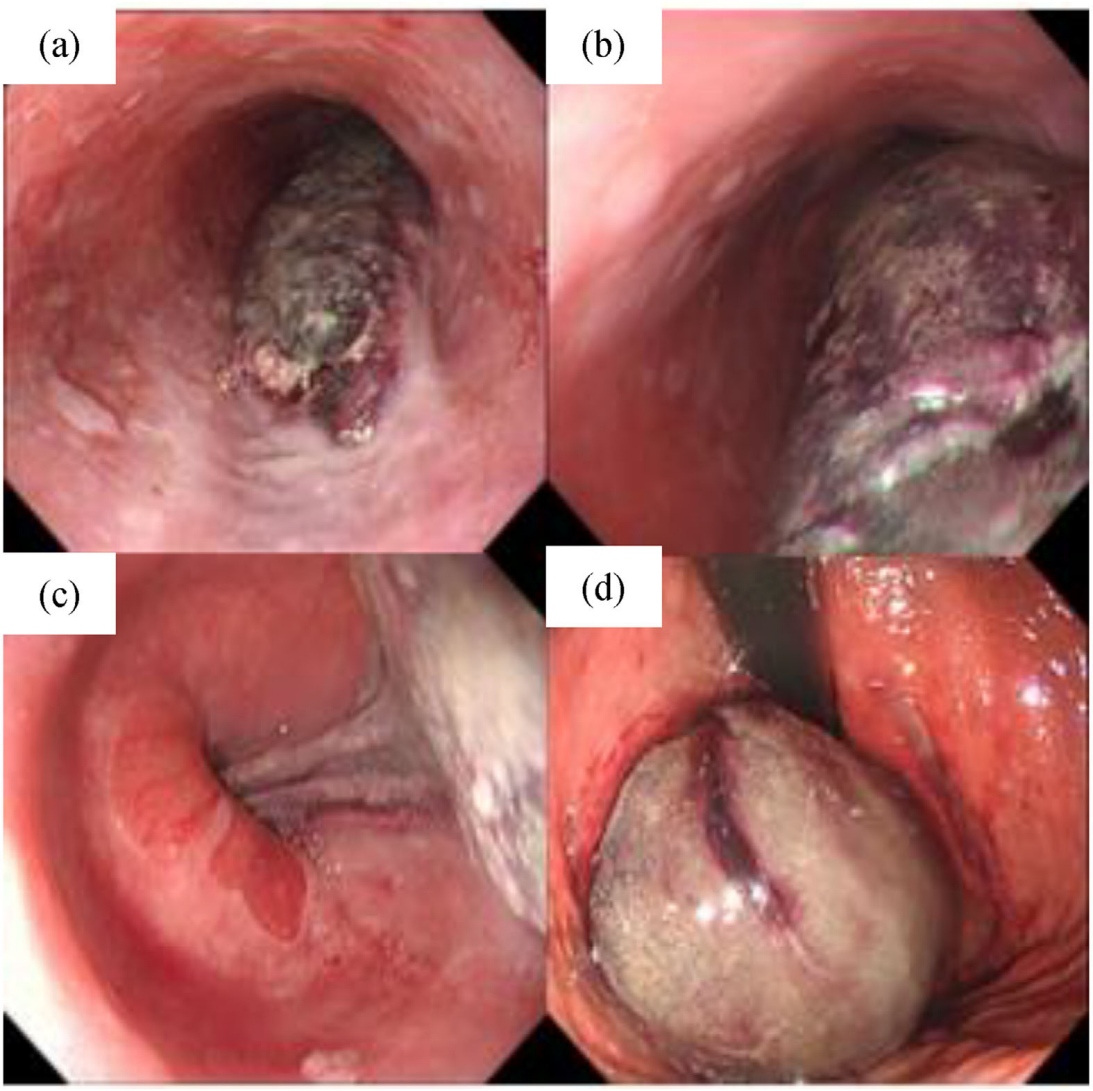
(a) On day 2, esophagogastroduodenoscopy revealed a reddish‐purple submucosal tumor‐like enhancement in the thoracic esophagus (30 cm from the oral cavity), which was thought to be a hematoma. (b) The hematoma was continuous toward the stomach, and the esophageal wall was partially narrowed. (c) However, passage of the scope was possible and the hematoma extended beyond the esophagogastric junction into the stomach. (d) The hematoma was observed from the fundus to the upper body of the stomach.

Treatment consisted of fasting, rehydration, and oral administration of vonoprazan 20 mg. On day 2, esophagogastroduodenoscopy (EGD) revealed a reddish‐purple submucosal tumor‐like enhancement extending vertically from the thoracic esophagus to the isthmus (Figure 2). Furthermore, magnetic resonance imaging (MRI) showed high signal intensity on T1‐weighted images and low signal intensity on T2‐weighted images in the same area, leading to the diagnosis of acute to subacute gastroesophageal submucosal hematoma (Figure 1c,d). Although endoscopic ultrasonography was contemplated, it was not performed due to the potential risk of hematoma rupture from contact with the scope, resulting in substantial bleeding.

**FIGURE 3:**
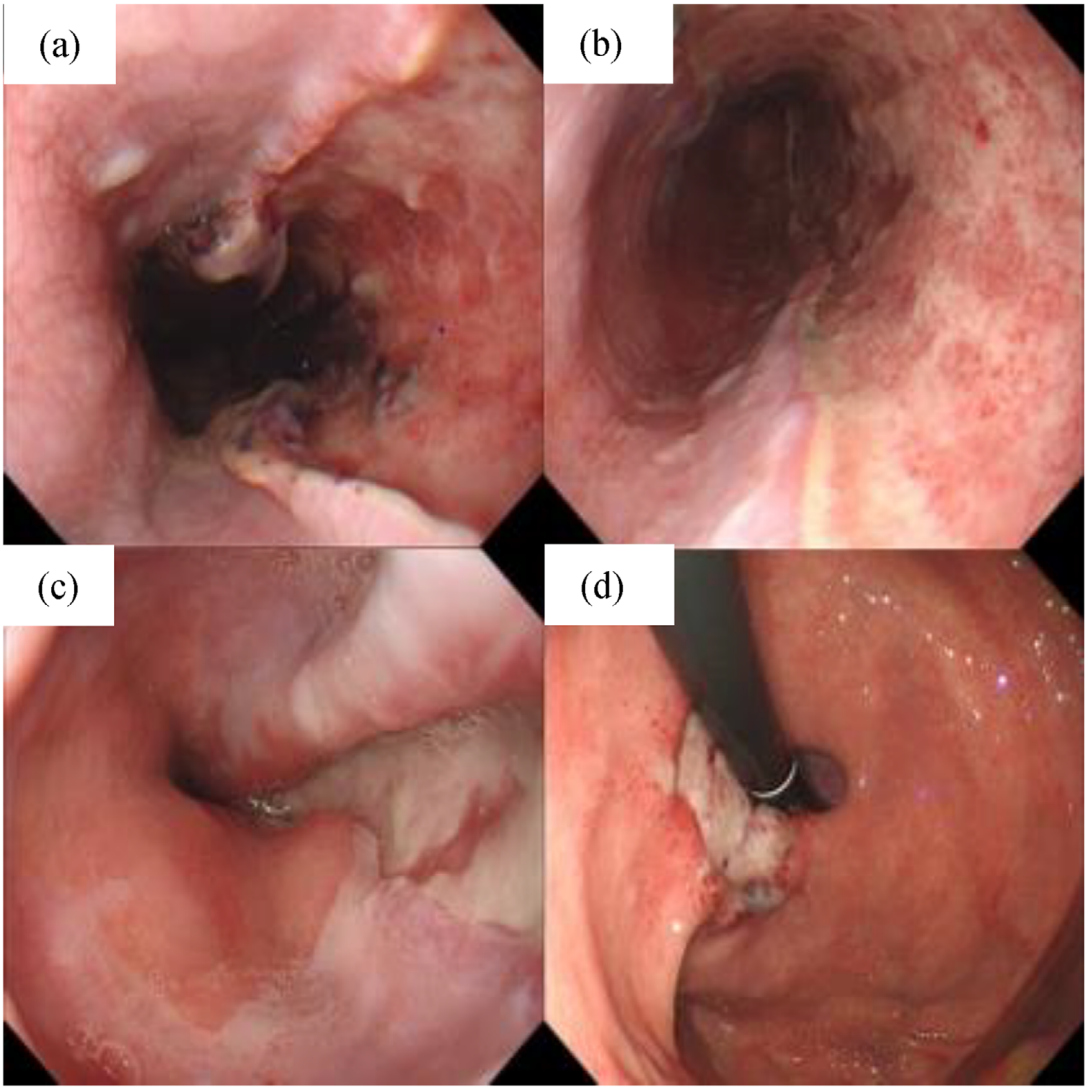
(a) On day 9, esophagogastroduodenoscopy showed that the esophageal hematoma had resolved and an ulcer had formed. (b) The hematoma at the site of the narrowing had also disappeared and no stricture was observed. (c) The ulcer extended from the esophagus to the stomach. (d) The intragastric hematoma had also disappeared and an ulcer had formed.

**FIGURE 4:**
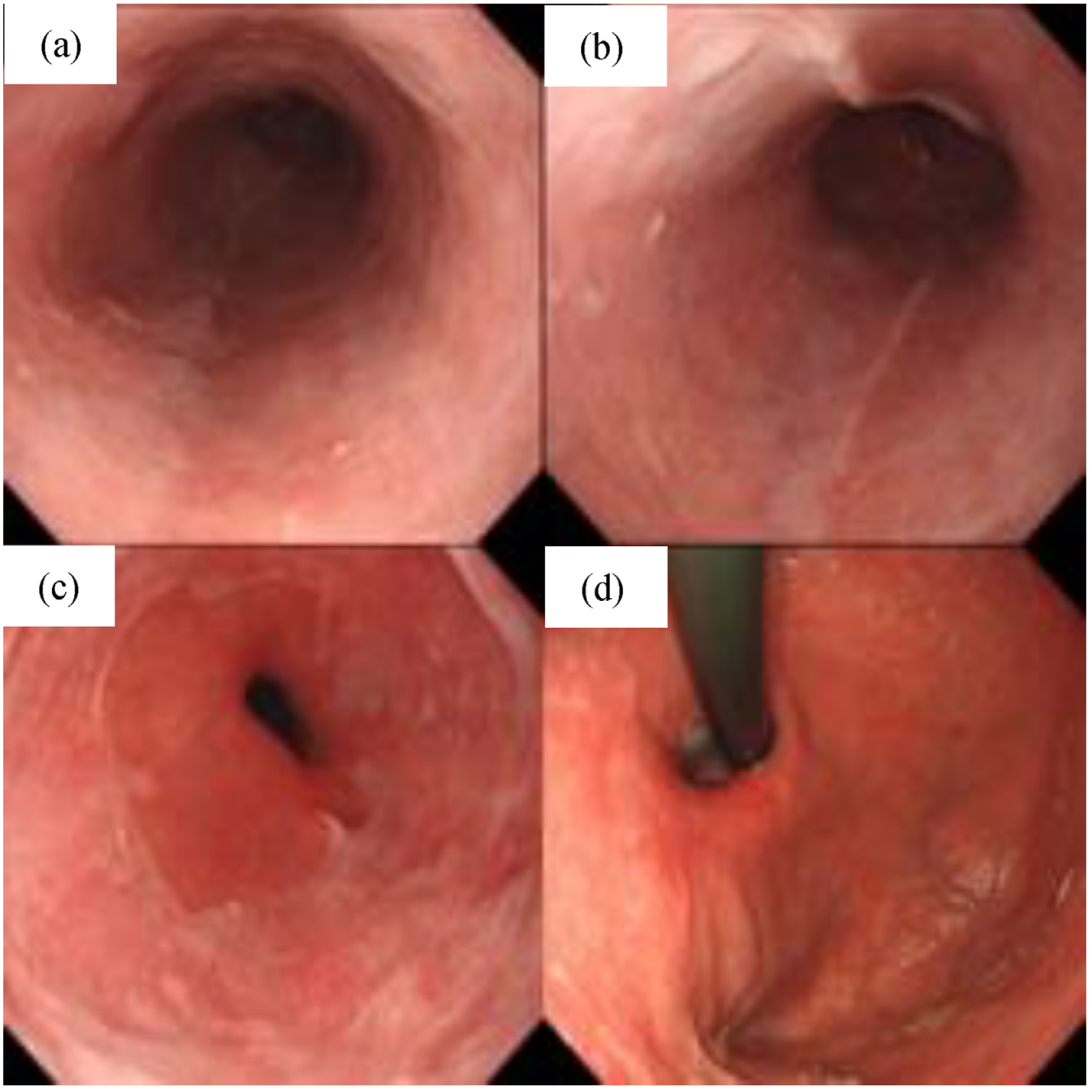
(a–c) Three months later, esophagogastroduodenoscopy revealed that the base of the esophageal ulcer was covered by normal mucosa and scarred. (d) The ulcer in the area from the fundus to the upper body of the stomach was also scarred and had a good healing course.

Because there was no evidence of esophageal obstruction, massive bleeding, or rapid hematoma enlargement on EGD, no endoscopic procedures such as incisional drainage were undertaken. The patient was managed conservatively with fasting and rehydration. After hospitalization, the precordial pain gradually abated, and subsequent blood tests showed no progression of anemia. A follow‐up EGD performed on day 9 showed the disappearance of the hematoma and the appearance of an ulcer at the site of the previous hematoma (Figure 3). On day 10, the patient resumed oral intake and was subsequently discharged from the hospital on day 16 as he remained asymptomatic with no signs of anemia progression in the blood tests. A 3‐month follow‐up EGD showed scarring of the ulcer base, confirming the healing process (Figure 4).

## DISCUSSION

Spontaneous occurrence of this condition in patients without hematologic disorders, coagulation abnormalities, or other predisposing factors to bleeding is rare. A comprehensive search on PubMed using the keywords “esophageal, submucosal, and hematoma” or “esophageal, intramural, and hematoma” yielded a total of 113 documented cases of esophageal submucosal hematoma diagnosed between 2002 and 2022. Of these, only 12 cases manifested spontaneously without any predisposition to bleeding (Table S1).

The etiology of gastroesophageal submucosal hematoma can be divided into spontaneous and traumatic types. In the spontaneous type, nausea, vomiting, and coagulopathy cause a sudden rise in pressure, leading to the rupture of blood vessels in the submucosa, resulting in hemorrhage and hematoma. In the traumatic type, food ingestion, endoscopy, and bougies can be the cause.^1^ Moreover, the use of antiplatelet and anticoagulant medications increases the risk of this condition.^2^ In this case, there was no history of food ingestion that could trigger the onset of the disease, such as consumption of bony fish or hot substances. The increase in esophageal pressure during vomiting may have precipitated the development of the condition. Although the patient had no hematologic or coagulation disorders, he had hypertension and diabetes mellitus, both of which can contribute to the development of atherosclerosis and may have predisposed him to the disease.

Typical symptoms of this condition include sudden retrosternal chest pain, dysphagia, and hematemesis. However, only 35% of patients experience all three symptoms, with the majority experiencing a combination of two of these symptoms.^3^ CT, MRI, and EGD are valuable diagnostic tools. CT generally shows symmetric or asymmetric esophageal wall thickening along with a well‐defined, non‐enhancing, hyperattenuating intraluminal mass extending along the esophageal wall.^4^ MRI typically shows a mass with intermediate signal intensity on T1‐ and T2‐weighted images. In addition, MRI enables visualization of hematoma expansion in different planes, which helps differentiate esophageal lesions from other mediastinal abnormalities.^5^ EGD typically shows a large blue or purplish mobile lesion descending along the posterior esophagus, sometimes accompanied by mucosal erosion, ulceration, or necrosis in cases where the submucosal lesion is extensive.^6^ The present case had all these characteristic imaging features and met the criteria for a typical idiopathic gastroesophageal submucosal hematoma.

Differential diagnoses for this condition encompass acute cardiovascular diseases such as acute myocardial infarction and aortic dissection, Mallory‐Weiss syndrome, and Boerhaave's syndrome. Differentiation between these disorders is based on medical history, including episodes of hematemesis and dysphagia, and findings on physical examination, EGD, CT, and electrocardiography. Treatment modalities comprise fasting, acid suppression, hemostasis, esophageal and gastric mucosal protection, and supportive care. Hematomas usually resolve within two to three weeks, and the prognosis is favorable.^7^ Esophageal obstruction due to hematoma is rare, and when it does transpire, the hematoma can be evacuated by endoscopic or surgical means.^8^, ^9^ In the majority of cases, esophageal obstruction improves with conservative management such as fasting and acid suppression.

In this particular case, the patient's vital signs were stable on presentation, and the hematoma was not extensive enough to cause dysphagia or esophageal obstruction during endoscopy. Therefore, a conservative approach was taken, which yielded a positive outcome for the patient. When a patient presents with abrupt precordial pain, hematemesis, and dysphagia, the possibility of this condition should be considered.

## CONFLICT OF INTEREST STATEMENT

None.

## Supporting information

Table S1 A case of esophageal submucosal hematoma in the absence of antithrombotic medication or predisposition to bleeding (12 cases).
