# Supplementary material for: A case of idiopathic gastroesophageal submucosal hematoma in a patient with no predisposition to bleeding
Source: DEN Open. 2023 Aug 21;4(1):e284. doi: 10.1002/deo2.284 (PMC10442609; doi:10.1002/deo2.284)
Supplement: Supplementary file 1 — Table S1 A case of esophageal submucosal hematoma in the absence of antithrombotic medication or predisposition to bleeding (12 cases). [file DEO2-4-e284-s001.pptx]

## Slide 1
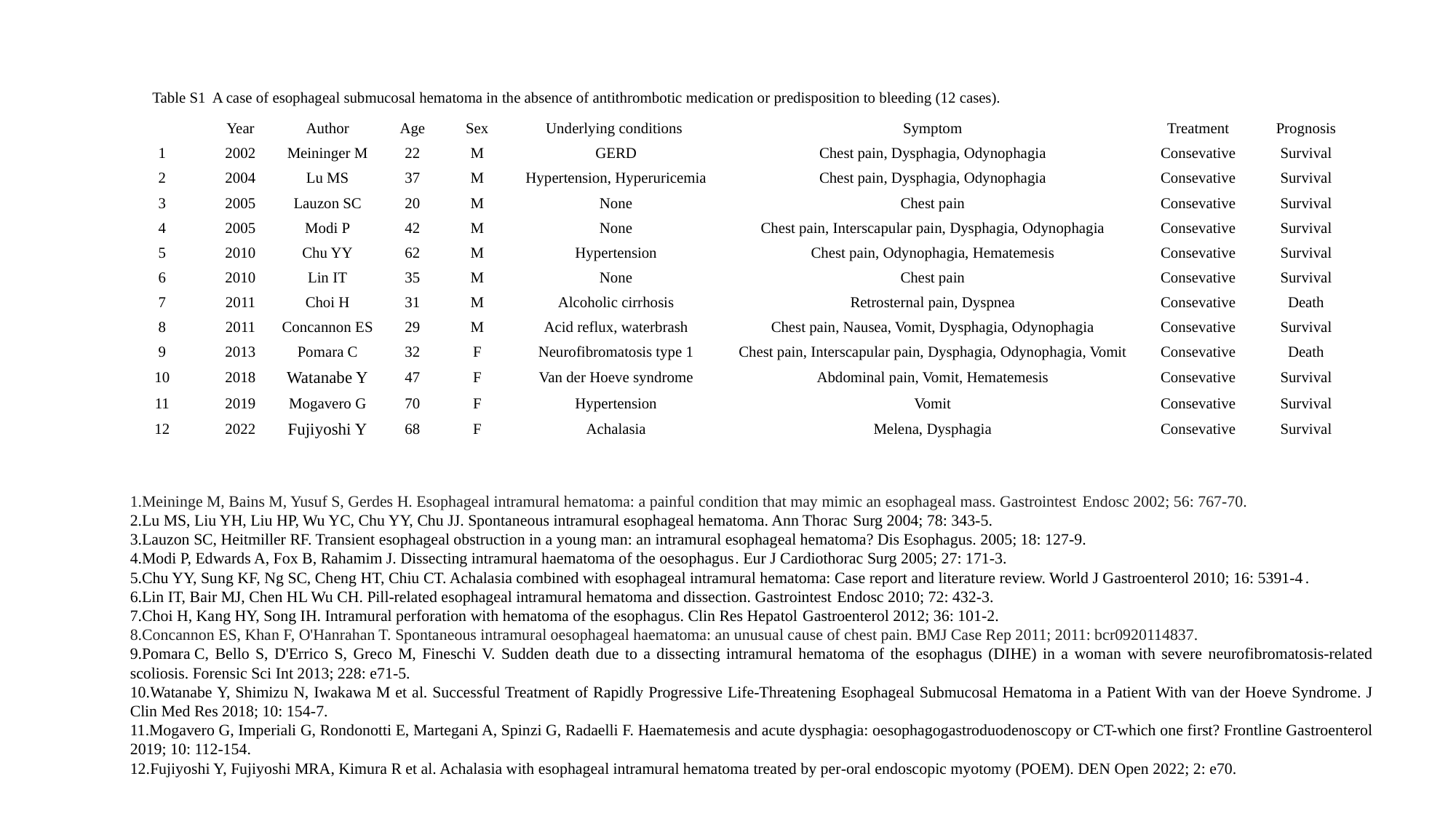

Table S1 A case of esophageal submucosal hematoma in the absence of antithrombotic medication or predisposition to bleeding (12 cases).
| | Year | Author | Age | Sex | Underlying conditions | Symptom | Treatment | Prognosis |
| --- | --- | --- | --- | --- | --- | --- | --- | --- |
| 1 | 2002 | Meininger M | 22 | M | GERD | Chest pain, Dysphagia, Odynophagia | Consevative | Survival |
| 2 | 2004 | Lu MS | 37 | M | Hypertension, Hyperuricemia | Chest pain, Dysphagia, Odynophagia | Consevative | Survival |
| 3 | 2005 | Lauzon SC | 20 | M | None | Chest pain | Consevative | Survival |
| 4 | 2005 | Modi P | 42 | M | None | Chest pain, Interscapular pain, Dysphagia, Odynophagia | Consevative | Survival |
| 5 | 2010 | Chu YY | 62 | M | Hypertension | Chest pain, Odynophagia, Hematemesis | Consevative | Survival |
| 6 | 2010 | Lin IT | 35 | M | None | Chest pain | Consevative | Survival |
| 7 | 2011 | Choi H | 31 | M | Alcoholic cirrhosis | Retrosternal pain, Dyspnea | Consevative | Death |
| 8 | 2011 | Concannon ES | 29 | M | Acid reflux, waterbrash | Chest pain, Nausea, Vomit, Dysphagia, Odynophagia | Consevative | Survival |
| 9 | 2013 | Pomara C | 32 | F | Neurofibromatosis type 1 | Chest pain, Interscapular pain, Dysphagia, Odynophagia, Vomit | Consevative | Death |
| 10 | 2018 | Watanabe Y | 47 | F | Van der Hoeve syndrome | Abdominal pain, Vomit, Hematemesis | Consevative | Survival |
| 11 | 2019 | Mogavero G | 70 | F | Hypertension | Vomit | Consevative | Survival |
| 12 | 2022 | Fujiyoshi Y | 68 | F | Achalasia | Melena, Dysphagia | Consevative | Survival |
Meininge M, Bains M, Yusuf S, Gerdes H. Esophageal intramural hematoma: a painful condition that may mimic an esophageal mass. Gastrointest Endosc 2002; 56: 767-70.
Lu MS, Liu YH, Liu HP, Wu YC, Chu YY, Chu JJ. Spontaneous intramural esophageal hematoma. Ann Thorac Surg 2004; 78: 343-5.
Lauzon SC, Heitmiller RF. Transient esophageal obstruction in a young man: an intramural esophageal hematoma? Dis Esophagus. 2005; 18: 127-9.
Modi P, Edwards A, Fox B, Rahamim J. Dissecting intramural haematoma of the oesophagus. Eur J Cardiothorac Surg 2005; 27: 171-3.
Chu YY, Sung KF, Ng SC, Cheng HT, Chiu CT. Achalasia combined with esophageal intramural hematoma: Case report and literature review. World J Gastroenterol 2010; 16: 5391-4.
Lin IT, Bair MJ, Chen HL Wu CH. Pill-related esophageal intramural hematoma and dissection. Gastrointest Endosc 2010; 72: 432-3.
Choi H, Kang HY, Song IH. Intramural perforation with hematoma of the esophagus. Clin Res Hepatol Gastroenterol 2012; 36: 101-2.
Concannon ES, Khan F, O'Hanrahan T. Spontaneous intramural oesophageal haematoma: an unusual cause of chest pain. BMJ Case Rep 2011; 2011: bcr0920114837.
Pomara C, Bello S, D'Errico S, Greco M, Fineschi V. Sudden death due to a dissecting intramural hematoma of the esophagus (DIHE) in a woman with severe neurofibromatosis-related scoliosis. Forensic Sci Int 2013; 228: e71-5.
Watanabe Y, Shimizu N, Iwakawa M et al. Successful Treatment of Rapidly Progressive Life-Threatening Esophageal Submucosal Hematoma in a Patient With van der Hoeve Syndrome. J Clin Med Res 2018; 10: 154-7.
Mogavero G, Imperiali G, Rondonotti E, Martegani A, Spinzi G, Radaelli F. Haematemesis and acute dysphagia: oesophagogastroduodenoscopy or CT-which one first? Frontline Gastroenterol 2019; 10: 112-154.
Fujiyoshi Y, Fujiyoshi MRA, Kimura R et al. Achalasia with esophageal intramural hematoma treated by per‐oral endoscopic myotomy (POEM). DEN Open 2022; 2: e70.
